# Supplementary material for: Chemical signals act as the main reproductive barrier between sister and mimetic Heliconius butterflies
Source: Proc Biol Sci. 2020 May 6;287(1926):20200587. doi: 10.1098/rspb.2020.0587 (PMC7282924; doi:10.1098/rspb.2020.0587)
Supplement: Supplementary Information [file rspb20200587supp1.pdf]

SUPPLEMENTARY INFORMATION
Proceedings of the Royal Society B: Biological Sciences
DOI: 10.1098/rspb.2020.0587

Chemical signals act as the main reproductive barrier between sister and mimetic *Heliconius* butterflies

González-Rojas, M.F.[1], Darragh, K.[2], Robles, J[3]., Linares, M.[1], Schulz, S[4]., McMillan, W.O[5]., Jiggins, C.D[2]., Pardo-Diaz, C[1]., Salazar, C[1].

Table S1. Samples included in the quantification analysis of wing phenotype 3
Table S2. Female behavioural response towards males with normal (control) and altered (treatment) wing phenotype. 6
Table S3. Female behavioural responses in triads that tested female preference for males "perfumed" with a hexanic extract from five males either of *H. melpomene malleti* or *H. timareta florencia*. 7
Table S4. Amount (ng) of compounds that remained in the wings of perfumed males before evaporation. 8
Table S5. Behavioural response of F_1 and backcross females towards pure males of *H. melpomene malleti* and *H. timareta florencia*. 11
Table S6. Compounds identified in wing androconia's extracts of males of *H. melpomene malleti*, *H. timareta florencia*, F_1 and backcross. 12
Table S7. Compounds identified in the abdominal glands' extracts of males of *H. melpomene malleti*, *H. timareta florencia*, F_1 and backcross 15
Table S8. Probability of mating in no-choice experiments 19
Figure S1. Map showing the geographic distribution and wing phenotype of *H. melpomene malleti* and *H. timareta florencia*. 20
Figure S2. Location of landmarks (LM) coordinates on the forewings and hindwings of *H. melpomene malleti* and *H. timareta florencia* 21
Figure S3. Species wing size and shape 22
Figure S4. (A) Shape variation of forewings of *H. melpomene malleti* (light grey) and *H. timareta florencia* (dark grey) 23
Figure S5. Colour pattern comparison between the two species 24

Figure S6. Mate choice triads testing the importance of wing colour pattern in mate preference. 26
Figure S7. Amount (ng) of compounds that remained in the wings of perfumed males before evaporation 27
Figure S8. Mate choice triads testing behavioural responses in F1 and backcross (BC) females 28
Figure S9. Species differences in male androconia extracts 29
Figure S10. Cluster analysis based on Euclidian distance of compound composition in the wing androconia of males of *H. melpomene malleti*, *H. timareta florencia*, F_1 and backcrosses (BC) 30
Figure S11. Chromatogram patterns obtained from androconial extracts of F_1 and backcross males 31
Figure S12. Chromatogram pattern of the abdominal gland bouquet of males. 32
Figure S13. Cluster analysis based on Euclidian distance of the compound composition of the wing androconia of males of *H. melpomene malleti*, *H. timareta florencia*, F_1 and backcrosses (BC) 33
Figure S14. Chromatogram patterns obtained from abdominal gland bouquet of F_1 and backcross males 34
REFERENCES 35

Table S1. Samples included in the quantification analysis of wing phenotype. A total of 89 individuals were used. The wings were obtained from "Colección de Artrópodos de la Universidad del Rosario (CAUR229)". Wild individuals of *H. timareta florencia* and *H. melpomene malleti* were collected in the localities Sucre and Doraditas in Colombia (01°48'12" N - 75°39'19" W, 1200 m and 01°42'39" N - 75°42'32" W, 1400 m). The last column specifies the analysis in which each specimen was used. D: dorsal; V: ventral; HW: hindwing; FW: forewing.

ID Collection	ID Wing Scan	Taxon	Locality	Analysis in which the sample was used
M54	LGE-WS-00351	*H. t. florencia*	Quebrada_Las_Doraditas	D-HW; D-FW
M63	LGE-WS-00349	*H. t. florencia*	Finca_Piñacue	D-HW; D-FW; V-HW; V-FW
M64	LGE-WS-00350	*H. t. florencia*	Finca_Piñacue	D-HW; D-FW; V-HW; V-FW
M244	LGE-WS-00373	*H. m. malleti*	Florencia	D-HW; D-FW; V-HW; V-FW
M253	LGE-WS-00375	*H. m. malleti*	Florencia_Sucre	D-HW; D-FW; V-HW; V-FW
M255	LGE-WS-00326	*H. t. florencia*	Florencia_Sucre	D-HW; D-FW
M257	LGE-WS-00346	*H. t. florencia*	Florencia_Sucre	D-HW; D-FW; V-HW; V-FW
M259	LGE-WS-00325	*H. t. florencia*	Florencia_Sucre	D-HW; D-FW
M415	LGE-WS-00397	*H. m. malleti*	Florencia_Sucre	D-HW; D-FW; V-HW; V-FW
M418	LGE-WS-00305	*H. t. florencia*	Florencia_Sucre	D-HW; D-FW; V-HW; V-FW
M426	LGE-WS-00361	*H. m. malleti*	Florencia_Sucre	D-HW; D-FW; V-HW; V-FW
M433	LGE-WS-00390	*H. m. malleti*	Florencia_Sucre	V-HW; V-FW
M434	LGE-WS-00363	*H. m. malleti*	Florencia_Sucre	V-HW; V-FW
M451	LGE-WS-00304	*H. t. florencia*	Florencia_Sucre	D-HW; D-FW; V-HW; V-FW
M462	LGE-WS-00337	*H. t. florencia*	Florencia_Sucre	D-HW; D-FW; V-HW; V-FW
M468	LGE-WS-00383	*H. m. malleti*	Florencia_Sucre	D-HW; D-FW; V-HW; V-FW
M471	LGE-WS-00307	*H. t. florencia*	Florencia_Sucre	D-HW; D-FW;
M472	LGE-WS-00311	*H. t. florencia*	Florencia_Sucre	D-HW; D-FW; V-HW; V-FW
M474	LGE-WS-00359	*H. m. malleti*	Florencia_Sucre	V-HW; V-FW
M583	LGE-WS-00367	*H. m. malleti*	Florencia	D-HW; D-FW; V-HW; V-FW
M584	LGE-WS-00357	*H. m. malleti*	Florencia_Sucre	D-HW; D-FW
M587	LGE-WS-00306	*H. t. florencia*	Florencia_Sucre	D-HW; D-FW; V-HW; V-FW
M588	LGE-WS-00310	*H. t. florencia*	Florencia_Sucre	D-HW; D-FW; V-HW; V-FW
M589	LGE-WS-00370	*H. m. malleti*	Florencia_Sucre	D-HW; D-FW; V-HW; V-FW
M590	LGE-WS-00395	*H. m. malleti*	Florencia_Sucre	D-HW; D-FW; V-HW; V-FW
M592	LGE-WS-00394	*H. m. malleti*	Florencia_Sucre	D-HW; D-FW; V-HW; V-FW
M593	LGE-WS-00309	*H. t. florencia*	Florencia_Sucre	D-HW; D-FW; V-HW; V-FW
M594	LGE-WS-00368	*H. m. malleti*	Quebrada_Las_Doraditas	D-HW; D-FW; V-HW; V-FW
M595	LGE-WS-00318	*H. t. florencia*	Florencia_Sucre	D-HW; D-FW; V-HW; V-FW
M596	LGE-WS-00308	*H. t. florencia*	Florencia_Sucre	D-HW; D-FW; V-HW; V-FW
M598	LGE-WS-00377	*H. m. malleti*	Florencia_Sucre	D-HW; D-FW; V-HW; V-FW

M602	LGE-WS-00303	*H. t. florencia*	Florencia_Sucre	D-HW; D-FW; V-HW; V-FW
M606	LGE-WS-00352	*H. m. malleti*	Florencia_Sucre	D-HW; D-FW; V-HW; V-FW
M607	LGE-WS-00317	*H. t. florencia*	Florencia_Sucre	D-HW; D-FW; V-HW; V-FW
M610	LGE-WS-00379	*H. m. malleti*	Florencia_Sucre	D-HW; D-FW; V-HW; V-FW
M611	LGE-WS-00316	*H. t. florencia*	Florencia_Sucre	D-HW; D-FW; V-HW; V-FW
M612	LGE-WS-00319	*H. t. florencia*	Florencia_Sucre	D-HW; D-FW; V-HW; V-FW
M614	LGE-WS-00355	*H. m. malleti*	Florencia_Sucre	D-HW; D-FW; V-HW; V-FW
M616	LGE-WS-00334	*H. t. florencia*	Florencia_Sucre	D-HW; D-FW; V-HW; V-FW
M618	LGE-WS-00324	*H. t. florencia*	Florencia_Sucre	D-HW; D-FW
M620	LGE-WS-00302	*H. t. florencia*	Florencia_Sucre	D-HW; D-FW; V-HW; V-FW
M622	LGE-WS-00332	*H. t. florencia*	Florencia_Sucre	D-HW; D-FW
M1009	LGE-WS-00333	*H. t. florencia*	Florencia_Sucre	D-HW; D-FW; V-HW; V-FW
M1010	LGE-WS-00313	*H. t. florencia*	Florencia_Sucre	D-HW; D-FW; V-HW; V-FW
M1016	LGE-WS-00378	*H. m. malleti*	Florencia_Sucre	D-HW; D-FW; V-HW; V-FW
M1074	LGE-WS-00315	*H. t. florencia*	Florencia_Sucre	D-HW; D-FW; V-HW; V-FW
M1075	LGE-WS-00314	*H. t. florencia*	Florencia_Sucre	D-HW; D-FW; V-HW; V-FW
M1079	LGE-WS-00348	*H. t. florencia*	Florencia_Sucre	D-HW; D-FW; V-HW; V-FW
M1084	LGE-WS-00328	*H. t. florencia*	Florencia_Sucre	D-HW; D-FW; V-HW; V-FW
M1085	LGE-WS-00329	*H. t. florencia*	Florencia_Sucre	D-HW; D-FW
M1094	LGE-WS-00330	*H. t. florencia*	Florencia_Sucre	D-HW; D-FW; V-HW; V-FW
M1098	LGE-WS-00396	*H. m. malleti*	Florencia_Sucre	D-HW; D-FW; V-HW; V-FW
M1196	LGE-WS-00354	*H. m. malleti*	Florencia_Paraiso	D-HW; D-FW; V-HW; V-FW
M1283	LGE-WS-00353	*H. m. malleti*	Florencia_Paraiso	D-HW; D-FW; V-HW; V-FW
M1288	LGE-WS-00364	*H. m. malleti*	Florencia_Paraiso	V-HW; V-FW
M1321	LGE-WS-00365	*H. m. malleti*	Florencia_Paraiso	D-HW; D-FW; V-HW; V-FW
M1441	LGE-WS-00381	*H. m. malleti*	Florencia_Sucre	D-HW; D-FW; V-HW; V-FW
M1507	LGE-WS-00389	*H. m. malleti*	Florencia_Paraiso	V-HW; V-FW
M1511	LGE-WS-00387	*H. m. malleti*	Florencia_Paraiso	D-HW; D-FW; V-HW; V-FW
M1512	LGE-WS-00386	*H. m. malleti*	Florencia_Paraiso	D-HW; D-FW; V-HW; V-FW
M1514	LGE-WS-00384	*H. m. malleti*	Florencia_Paraiso	D-HW; D-FW; V-HW; V-FW
M1522	LGE-WS-00385	*H. m. malleti*	Florencia_Paraiso	D-HW; D-FW; V-HW; V-FW
M1754	LGE-WS-00331	*H. t. florencia*	Florencia_Sucre	D-HW; D-FW; V-HW; V-FW
M1757	LGE-WS-00376	*H. m. malleti*	Florencia_Sucre	D-HW; D-FW; V-HW; V-FW
M1758	LGE-WS-00345	*H. t. florencia*	Florencia_Sucre	D-HW; D-FW; V-HW; V-FW
M1767	LGE-WS-00391	*H. m. malleti*	Florencia_Sucre	D-HW; D-FW; V-HW; V-FW
M1769	LGE-WS-00343	*H. t. florencia*	Florencia_Sucre	D-HW; D-FW; V-HW; V-FW
M1770	LGE-WS-00388	*H. m. malleti*	Florencia_Sucre	V-HW; V-FW
M1771	LGE-WS-00344	*H. t. florencia*	Florencia_Sucre	D-HW; D-FW; V-HW; V-FW
M1772	LGE-WS-00321	*H. t. florencia*	Florencia_Sucre	D-HW; D-FW; V-HW; V-FW
M1773	LGE-WS-00369	*H. m. malleti*	Florencia_Sucre	D-HW; D-FW; V-HW; V-FW
M1774	LGE-WS-00360	*H. m. malleti*	Florencia_Sucre	D-HW; D-FW; V-HW; V-FW
M1805	LGE-WS-00320	*H. t. florencia*	Florencia_Sucre	D-HW; D-FW; V-HW; V-FW
M1808	LGE-WS-00322	*H. t. florencia*	Florencia_Sucre	D-HW; D-FW
M1813	LGE-WS-00366	*H. m. malleti*	Quebrada_Las_Doraditas	D-HW; D-FW; V-HW; V-FW
M1814	LGE-WS-00399	*H. m. malleti*	Florencia_Sucre	D-HW; D-FW
M1817	LGE-WS-00323	*H. t. florencia*	Florencia_Sucre	V-HW; V-FW
M1823	LGE-WS-00356	*H. m. malleti*	Florencia_Paraiso	D-HW; D-FW; V-HW; V-FW
M1845	LGE-WS-00362	*H. m. malleti*	Florencia_Paraiso	D-HW; D-FW; V-HW; V-FW

M1846	LGE-WS-00336	*H. t. florencia*	Florencia_Sucre	D-HW; D-FW; V-HW; V-FW
M2347	LGE-WS-00393	*H. m. malleti*	Florencia_Paraiso	D-HW; D-FW; V-HW; V-FW
M2360	LGE-WS-00374	*H. m. malleti*	Florencia_Paraiso	D-HW; D-FW; V-HW; V-FW
M2408	LGE-WS-00382	*H. m. malleti*	Florencia_Paraiso	D-HW; D-FW; V-HW; V-FW
M3544	LGE-WS-00358	*H. m. malleti*	Florencia_Paraiso	D-HW; D-FW; V-HW; V-FW
M3765	LGE-WS-00347	*H. t. florencia*	Florencia_Sucre	D-HW; D-FW; V-HW; V-FW
M3767	LGE-WS-00339	*H. t. florencia*	Florencia_Sucre	D-HW; D-FW; V-HW; V-FW
M3874	LGE-WS-00341	*H. t. florencia*	Florencia_Sucre	D-HW; D-FW; V-HW; V-FW
M3875	LGE-WS-00327	*H. t. florencia*	Florencia_Sucre	D-HW; D-FW

Table S2. Female behavioural response towards males with normal (control) and altered (treatment) wing phenotype. Behaviours are classified as Acceptance or Rejection. The asterisk (*) indicates statistical significance (α=0.01) according to the GLMM.

Behaviour		*H. m. malleti*	*H. t. florencia*
Flutter	Acceptance	$\chi^2_{(1,326)}$=0.646, p>0.01	$\chi^2_{(1,216)}$=4.734, p>0.01
Fly towards	Acceptance	$\chi^2_{(1,326)}$=0.877, p>0.01	$\chi^2_{(1,216)}$=0.090, p>0.01
Slow flat	Acceptance	$\chi^2_{(1,326)}$=0.608, p>0.01	$\chi^2_{(1,216)}$=1.223, p>0.01
Wings open	Acceptance	$\chi^2_{(1,326)}$=2.005, p>0.01	$\chi^2_{(1,216)}$=6.905, p<0.01*
Abdomen exposed	Acceptance	$\chi^2_{(1,326)}$=0.215, p>0.01	$\chi^2_{(1,216)}$=0.067, p>0.01
Fly away	Rejection	$\chi^2_{(1,326)}$=2.353, p>0.01	$\chi^2_{(1,216)}$=1.474, p>0.01
Tucked up	Rejection	$\chi^2_{(1,326)}$=1.213, p>0.01	$\chi^2_{(1,216)}$=5.500, p>0.01
Erratic flutter	Rejection	$\chi^2_{(1,326)}$=1.723, p>0.01	$\chi^2_{(1,216)}$=2.495, p>0.01
Abdomen bent	Rejection	$\chi^2_{(1,326)}$=0.075, p>0.01	$\chi^2_{(1,216)}$=5.549, p>0.01

Table S3. Female behavioural responses in triads that tested female preference for males "perfumed" with a hexanic extract from five males either of *H. melpomene malleti* or *H. timareta florencia*. The asterisk (*) indicates statistical significance (α=0.01) according to the GLMM.

Behaviour		*H. m. malleti*	*H. t. florencia*
Flutter	Acceptance	$\chi^2_{(1=473)}$=51.113, p<0.01*	$\chi^2_{(1,354)}$=25.476, p<0.01*
Fly towards	Acceptance	$\chi^2_{(1,473)}$=75.612, p<0.01*	$\chi^2_{(1,354)}$=32.715, p<0.01*
Slow flat	Acceptance	$\chi^2_{(1,473)}$=44.195, p<0.01*	$\chi^2_{(1,354)}$=48.501, p<0.01*
Wings open	Acceptance	$\chi^2_{(1,473)}$=139.04, p<0.01*	$\chi^2_{(1,354)}$=93.902, p<0.01*
Abdomen exposed	Acceptance	$\chi^2_{(1,473)}$=90.039, p<0.01*	$\chi^2_{(1,354)}$=60.483, p<0.01*
Fly away	Rejection	$\chi^2_{(1,473)}$=44.263, p<0.01*	$\chi^2_{(1,354)}$=109.41, p<0.01*
Tucked up	Rejection	$\chi^2_{(1,473)}$=13.629, p<0.01*	$\chi^2_{(1,354)}$=8.1586, p<0.01*
Erratic flutter	Rejection	$\chi^2_{(1,473)}$=43.539, p<0.01*	$\chi^2_{(1,354)}$=75.385, p<0.01*
Abdomen bent	Rejection	$\chi^2_{(1,473)}$=27.207, p<0.01*	$\chi^2_{(1,354)}$=47.357, p<0.01*

Table S4. Amount (ng) of compounds that remained in the wings of perfumed males before evaporation. We quantified the presence of the perfume applied at the beginning of the experiment, at 1 minute, at 30 minutes and at 60 minutes after spreading the perfume. RI, retention index.

Name	RI	*H. melpomene malleti*				*H. timareta florencia*			
		Beginning	1 minute	30 minutes	60 minutes	Beginning	1 minute	30 minutes	60 minutes
Unknown	958.50	0.14	0.26	0.00	0.00	-	-	-	-
Limonene	1023.60	-	-	-	-	0.29	0.20	0.00	0.00
Phenylacetaldehyde	1036.10	-	-	-	-	0.17	0.00	0.00	0.00
Methyl salicylate	1187.20	-	-	-	-	1.44	1.37	0.00	0.03
Dodecane	1117.30	-	-	-	-	2.78	2.82	2.01	1.15
Unknown	1174.80	0.20	0.14	0.14	0.41	0.45	2.35	2.20	0.83
(Z)-3-Hexenyl isobutyrate	1233.30	19.22	17.46	0.00	0.00	-	-	-	-
Hexyl 3-methylbutyrate	1239.50	53.33	0.00	0.00	0.00	-	-	-	-
Unknown	1243.70	0.58	0.30	0.00	0.00	-	-	-	-
Alkane	1265.40	-	-	-	-	1.82	0.46	0.00	0.00
Tridecane	1300.00	15.16	18.51	15.01	5.62	7.67	7.24	4.64	1.72
Tetradecane	1302.40	-	-	-	-	0.67	0.70	0.64	0.62
5-Decanolide	1369.50	-	-	-	-	4.80	4.76	4.44	1.16
alpha-Copaene	1371.50	1.31	0.00	0.00	0.00	-	-	-	-
Dihydroactinidiolide	1391.70	17.67	17.35	14.13	8.36	14.40	13.25	7.91	6.63
Unknown	1394.80	0.90	0.73	0.41	0.00	-	-	-	-
Ethyl 4-ethoxybenzoate	1402.60	8.29	9.40	1.03	0.56	12.09	15.41	14.39	14.06
Homovanillyl alcohol	1412.10	1.56	1.68	1.16	0.45	0.25	0.00	0.00	0.00
Methy 4-hydroxybenzoate	1449.00	2.51	2.10	0.00	0.00	-	-	-	-
Methyl 3,4-	1464.10	8.97	7.53	5.88	3.25	-	-	-	-

dimethoxybenzoate									
Unknown	1470.10	0.92	0.57	0.56	0.30	-	-	-	-
Unknown	1488.30	-	-	-	-	0.11	0.00	0.00	0.00
Unknown	1495.20	0.10	0.16	0.12	0.06	-	-	-	-
Syringaaldehyde	1519.00	291.80	293.20	280.37	251.10	277.15	263.46	207.00	174.30
3,5-Dimethoxy 4-hydroxybenzyl alcohol	1565.00	-	-	-	-	7.17	0.00	0.00	0.00
Propyl 4-hydroxybenzoate	1614.60	-	-	-	-	2.70	0.00	0.00	0.00
Methyl 1H-indol-3-carboxylate	1663.10	-	-	-	-	0.24	0.28	0.26	0.07
Unknown	1715.80	1.22	2.10	0.00	0.64	-	-	-	-
Tricosene	1740.20	-	-	-	-	1.13	1.27	1.23	0.56
Hexadecanoic acid	1818.00	-	-	-	-	1.75	1.46	0.61	0.31
Octadecanal	1868.00	1094.90	826.74	202.60	130.10	-	-	-	-
Isopropyl Palmitate	1877.50	-	-	-	-	1.93	2.62	2.04	1.60
Unknown	1922.10	16.56	22.88	21.86	0.80	-	-	-	-
Heneicosene	1946.10	-	-	-	-	12.30	3.45	0.00	0.00
Heneicosane	1952.10	298.84	325.10	287.02	171.70	174.57	193.60	165.48	146.40
1-Octadecanol	1929.60	27.83	34.65	34.44	21.10	26.23	15.28	9.59	0.00
(Z)-11-Eicosenal	2031.20	231.99	63.82	59.68	32.72	3.86	2.48	0.00	0.00
Docosane	2044.40	0.28	4.70	4.41	0.00	-	-	-	-
Eicosanal	2058.90	26.43	35.88	31.85	0.00	-	-	-	-
Unknown	2109.90	-	-	-	-	0.50	0.00	0.00	0.00
Unknown	2135.80	1.54	1.99	1.77	0.64	0.20	0.25	0.23	0.11
Tricosane	2137.20	27.27	0.00	0.00	0.00	3.36	0.00	0.00	0.00
Isopropyl oleate	2187.70	-	-	-	-	0.00	0.03	0.00	0.00
Ethyl stearate	2190.40	-	-	-	-	1.77	1.24	0.00	0.00
(Z)-13-Docosenal	2217.80	23.67	0.00	0.00	0.00	-	-	-	-
Eicosane	2228.10	2.65	1.34	2.17	5.26	2.56	3.27	2.36	1.83

Heptacosane	2489.80	60.12	78.75	79.10	219.00	31.49	30.28	21.81	11.74
Octacosane	2569.30	-	-	-	-	0.40	0.00	0.00	0.00
Hexacosanal	2594.80	-	-	-	-	0.66	0.00	0.00	0.00
Nonacosane	2652.30	25.34	26.21	24.79	0.00	14.03	11.58	2.88	1.34
13,17-Dimethylnonacosane	2688.10	-	-	-	-	1.83	0.89	0.00	0.00
Ethyl benzoate	2686.30	-	-	-	-	0.31	0.32	0.30	0.00
Octacosanal	2740.80	12.51	15.64	13.83	0.00	28.55	24.31	11.60	1.18
Cholesterol	2807.00	286.19	229.63	128.30	0.00	236.60	92.43	87.18	38.70
Hentriacontane	2807.80	58.10	53.88	47.26	0.00	115.60	65.82	18.58	15.55
13,17-Dimethylhentriacontane	2835.80	12.95	10.76	1.64	0.00	3.93	2.65	1.74	0.00
2-Eicosyl-5-nonyltetrahydrofuran	3121.50	-	-	-	-	3.89	0.00	0.00	0.00

Table S5. Behavioural response of F_1 and backcross females towards pure males of *H. melpomene malleti* and *H. timareta florencia*. The asterisk (*) indicates statistical significance (α=0.01) according to the GLMM.

Behaviour		F_1 Females	Backcross Females
Flutter	Acceptance	$\chi^2_{(1,561)}$=2.103, p>0.01	$\chi^2_{(1,468)}$=0.1015, p>0.01
Fly towards	Acceptance	$\chi^2_{(1,561)}$=3.184, p>0.01	$\chi^2_{(1,468)}$=37.345, p<0.01*
Slow flat	Acceptance	$\chi^2_{(1,561)}$=50.916, p<0.01*	$\chi^2_{(1,468)}$=259.21, p<0.01*
Wings open	Acceptance	$\chi^2_{(1,561)}$=62.043, p<0.01*	$\chi^2_{(1,468)}$=57.215, p<0.01*
Abdomen exposed	Acceptance	$\chi^2_{(1,561)}$=10.650, p<0.01*	$\chi^2_{(1,468)}$=309.72, p<0.01*
Fly away	Rejection	$\chi^2_{(1,561)}$=0.600, p>0.01	$\chi^2_{(1,468)}$=97.105, p<0.01*
Tucked up	Rejection	$\chi^2_{(1,561)}$=35.489, p<0.01*	$\chi^2_{(1,468)}$=106.42, p<0.01*
Erratic flutter	Rejection	$\chi^2_{(1,561)}$=44.106, p<0.01*	$\chi^2_{(1,468)}$=72.130, p<0.01*
Abdomen bent	Rejection	$\chi^2_{(1,561)}$=24.640, p<0.01*	$\chi^2_{(1,468)}$=4.630, p>0.01

Table S6. Compounds identified in wing androconia's extracts of males of *H. melpomene malleti*, *H. timareta florencia*, F_1 and backcross. RI, retention index. Mean ± SD amounts in ng.

Name	RI	*H. melpomene malleti*	*H. timareta florencia*	F1	Backcrosses
		Mean ± SD	Mean ± SD	Mean ± SD	Mean± SD
Unknown	902.60	-	-	3.80 ± 4.91	0.78 ± 1.60
Dimethyl sulfone	916.00	-	-	0.76 ± 1.33	0.50 ± 1.99
Unknown	958.50	0.13 ± 0.76	0.43 ± 1.69	7.15 ± 4.04	11.25 ± 5.68
Limonene	1023.60	-	0.28 ± 0.52	-	-
Phenylacetaldehyde	1036.10	-	0.16 ± 0.77	-	-
Unknown	1037.60	-	-	1.50 ± 2.40	1.16 ± 1.85
Nonanal	1100.70	0.58 ± 1.51	1.47 ± 2.94	3.67 ± 4.97	-
Dodecane	1117.30	-	2.91 ± 5.72	-	-
Unknown	1174.80	-	0.06 ± 3.09	-	-
Methyl salicylate	1187.20	2.44 ± 2.51	1.39 ± 1.99	-	-
Decanal	1200.90	-	-	3.20 ± 8.32	-
(Z)-3-Hexenyl isobutyrate	1233.30	19.21 ± 14.04	-	-	-
Hexyl 3-methylbutyrate	1239.50	53.33 ± 144.25	-	-	-
Alkane	1265.40	-	1.76 ± 9.72	-	-
Tridecane	1300.00	15.16 ± 12.07	8.15 ± 9.15	-	-
Tetradecane	1302.90	-	0.92 ± 2.54	-	-
5-Decanolide	1369.90	-	4.65 ± 5.40	1.06 ± 1.88	0.12 ± 0.64
alpha-Copaene	1371.50	1.31 ± 1.88	-	-	-
Dihydroactinidiolide	1392.90	17.67 ± 11.54	13.96 ± 15.69	1.71 ± 1.84	0.93 ± 2.02
Ethyl 4-ethoxybenzoate	1403.40	8.28 ± 6.87	12.12 ± 16.23	8.28 ± 5.88	5.65 ± 6.65
Homovanillin alcohol	1413.10	1.55 ± 2.93	0.249 ± 0.78	0.27 ± 0.53	0.12 ± 0.47
6,10-Dimethyl 5,9-undecadien-2-one	1447.60	2.64 ± 3.10	-	-	-

Methy 4-hydroxybenzoate	1449.00	2.51 ± 4.83	-	-	-
Methyl 3,4-dimethoxybenzoate	1465.90	8.97 ± 18.25	-	-	-
Unknown	1470.10	0.19 ± 0.50	-	-	0.33 ± 0.65
Unknown	1488.30	-	0.09 ± 0.46	-	-
Unknown	1495.20	-	-	0.06 ± 0.22	-
Syringaaldehyde	1519.00	291.8 ± 234.84	268.75 ± 285.36	56.11 ± 62.01	34.18 ± 50.06
3,5-Dimethoxy 4-hydroxybenzyl alcohol	1565.00	-	6.95 ± 36.05	-	-
Propyl 4-hydroxybenzoate	1614.60	-	3.02 ± 5.43	-	-
Methyl 1H-indol-3-acetate	1663.10	-	0.23 ± 0.75	-	-
Unknown	1715.80	0.89 ± 1.51	-	2.60 ± 3.14	1.57 ± 1.77
Ethyl benzoate	1765.30	-	0.30 ± 0.89	0.19 ± 0.53	-
16-Hexadecanolide	1769.10	-	-	-	0.92 ± 4.01
Unknown	1795.00	-	3.72 ± 20.66	0.16 ± 0.40	-
Hexadecanoic acid	1826.50	-	2.76 ± 8.24	-	-
Octadecanal	1869.80	1094.91 ± 519.64	1.40 ± 5.84	19.25 ± 35.04	1.86 ± 5.38
Unknown	1873.80	-	-	0.14 ± 0.48	1.64 ± 4.85
Isopropyl palmitate	1878.30	-	1.86 ± 6.02	-	3.00 ± 8.28
Unknown	1923.40	16.56 ± 14.99	-	-	-
Unknown	1929.30	0.57 ± 2.42	-	1.02 ± 1.39	-
1-Octadecanol	1929.60	27.83 ± 33.07	29.85 ± 138.42	2.82 ± 6.48	-
Heneicosene	1946.10	0.47 ± 1.90	26.80 ± 104.03	5.97 ± 18.76	4.22 ± 11.20
Heneicosane	1953.70	298.83 ± 335.05	174.57 ± 225.37	132.51 ± 102.24	125.07 ± 122.00
(Z)-11-Eicosenal	2032.70	231.98 ± 255.99	4.39 ± 11.48	22.25 ± 20.38	3.75 ± 9.35
Docosane	2045.50	0.27 ± 0.99	6.25 ± 22.60	-	-
Eicosanal	2058.90	43.43 ± 53.98	-	1.46 ± 3.12	-
Unknown	2088.50	-	1.93 ± 11.14	-	-
Tricosene	2106.80	-	2.84 ± 16.35	0.20 ± 0.68	-
Unknown	2109.90	0.91 ± 4.15	-	0.28 ± 0.72	0.37 ± 0.86
Unknown	2135.80	1.53 ± 4.23	-	-	-
Tricosane	2137.20	27.27 ± 151.83	8.46 ± 31.72	-	-

Ethyl stearate	2190.40	-	1.71 ± 3.18	-	0.05 ± 0.19
(Z)-13-Docosenal	2217.80	23.66 ± 68.98	-	0.62 ± 2.08	-
Docosane	2227.90	-	0.19 ± 0.92	1.75 ± 3.75	0.36 ± 0.86
Eicosane	2228.10	2.63 ± 5.25	3.43 ± 7.06	0.10 ± 0.35	0.23 ± 0.56
Unknown	2321.80	0.10 ± 0.57	-	-	0.98 ± 2.78
Heptacosane	2411.00	56.11 ± 73.64	12.16 ± 39.18	23.78 ± 17.25	36.12 ± 30.35
Octacosane	2569.30	-	2.63 ± 15.11	-	-
Hexacosanal	2594.80	-	5.20 ± 26.32	-	-
Methylheptacosane	2596.10	15.88 ± 26.13	12.42 ± 39.54	4.24 ± 6.31	6.34 ± 15.06
Nonacosane	2652.30	25.34 ± 41.2	13.60 ± 25.45	31.43 ± 23.85	31.24 ± 38.05
Unknown	2687.70	-	0.39 ± 2.25	0.43 ± 1.44	-
13,17 Dimethylnonacosane	2688.10	-	1.77 ± 4.84	7.26 ± 7.19	1.17 ± 3.04
Octacosanal	2740.80	12.50 ± 21.93	28.82 ± 52.43	28.71 ± 28.59	23.6 ± 24.67
Unknown	2794.20	1.21 ± 3.23	-	0.15 ± 0.52	21.78 ± 69.18
Cholesterol	2807.00	286.19 ± 445.59	250.36 ± 219.16	218.74 ± 95.74	204.56 ± 135.52
Hentriacontane	2807.80	58.09 ± 100.82	118.43 ± 206.82	88.60 ± 39.46	52.32 ± 32.19
13,17-Dimethylhentriacontane	2835.80	12.95 ± 28.21	3.81 ± 17.50	18.13 ± 17.65	4.16 ± 8.97
2-Eicosyl-5-heptyltetrahydrofuran	2923.70	97.19 ± 257.04	-	-	-
2-Eicosyl-5-nonyltetrahydrofuran	3121.50	10.11 ± 56.30	4.69 ± 13.95	-	1.44 ± 7.23

Table S7. Compounds identified in the abdominal glands' extracts of males of *H. melpomene malleti*, *H. timareta florencia*, F_1 and backcross. RI, retention index. Mean ± SD amounts in ng.

Name	RI	*H. melpomene malleti*	*H. timareta florencia*	F1	Backcrosses
		Mean ± SD	Mean ± SD	Mean ± SD	Mean ± SD
Unknown	903.90	-	1.58 ± 3.99	7.08 ± 5.85	13.70 ± 12.24
Dimethyl sulfone	919.00	-	0.34 ± 1.22	-	-
(Z)-beta-Ocimene	1037.50	11899.84 ± 7633.07	-	142.08 ± 270.49	78.92 ± 243.28
Phenylacetonitril_Benzylcyanid	1039.80	-	62.59 ± 95.31	-	-
(E)-beta-Ocimene	1048.20	12096 ± 7193.57	-	2247.58 ± 3047.13	34.18 ± 75.04
2-sec-Butyl-3-methoxypyrazine	1170.10	-	74.80 ± 91.67	12.45 ± 16.86	55.28 ± 34.86
2-isobutyl-3-methoxy pyrazine	1177.70	0.02 ± 0.11	-	1.26 ± 1.51	2.43 ± 3.94
Methyl salicylate	1188.50	-	0.12 ± 0.54	1.74 ± 1.28	1.27 ± 1.68
5-Decanolide	1203.00	0.42 ± 2.24	0.18 ± 1.03	-	-
Dihydroedulan II	1284.90	43.92 ± 40.09	5.52 ± 14.98	1.82 ± 2.26	-
Tridecane	1300.00	1.33 ± 1.91	0.44 ± 0.87	-	-
Nonadecane	1302.70	554.93 ± 1403.34	721.14 ± 3082.59	-	-
alpha-Copaene	1374.10	0.02 ± 0.14	2.53 ± 6.81	-	-
GC-EAD active compound	1395.20	264.14 ± 333.29	622.68 ± 661.95	122.10 ± 124.78	47.67 ± 66.71
Dihydroactinidiolide	1517.30	0.09 ± 0.48	21.34 ± 120.35	-	-
Ethyl 4-ethoxybenzoate	1520.20	8.81 ± 7.73	18.26 ± 40.40	9.51 ± 6.44	6.02 ± 7.69
Homovanillyl alcohol	1534.00	1.43 ± 2.86	1.40 ± 4.25	0.18 ± 0.48	1.38 ± 2.72
Unknown	1542.90	0.18 ± 1.00	0.49 ± 2.54	3.83 ± 5.72	0.55 ± 1.05
Heptadecane	1700.40	155.78 ± 602.03	0.13 ± 0.76	0.24 ± 0.82	-
Benzyl_salicylate	1708.20	0.34 ± 0.84	-	-	-
Unknown	1712.00	-	23.37 ± 69.08	0.76 ± 1.33	-
14-Tetradecanolide	1721.50	5.86 ± 14.98	30.18 ± 62.64	40.03 ± 48.57	9.77 ± 15.12

Hexadecatrienolide	1726.10	-	3.14 ± 6.25	-	-
9_11-Hexadecadien-11-olide	1734.70	-	13.31 ± 25.54	-	-
Ethyl benzoate	1764.60	6.86 ± 14.56	11.54 ± 20.45	-	-
Unknown	1771.70	-	1.06 ± 3.42	32.52 ± 50.73	-
Macrolide	1773.70	0.5423 ± 2.86	0.81 ± 2.44	-	0.34 ± 1.78
(Z2_Z4)-C16-15-olide	1806.60	-	1.75 ± 6.20	-	-
Hexadecen-11-olide	1819.30	-	12.32 ± 16.05	-	-
Octadecatrienolide	1823.40	32.10 ± 32.03	26.36 ± 36.51	-	-
Hexadecenolide	1857.00	11.18 ± 24.31	126.3 ± 214.21	6.04 ± 11.35	13.09 ± 23.92
Macrolide	1923.60	6.98 ± 36.97	2.85 ± 10.88	-	0.45 ± 2.33
Heptadecanal	1923.70	-	-	8.64 ± 12.30	12.93 ± 22.66
16-Hexadecanolide	1924.80	-	124.38 ± 160.04	4.16 ± 11.61	-
Brassicalactone	1960.90	-	278.62 ± 496.97	11.79 ± 22.16	20.40 ± 50.00
Octadecen-11-olide	2002.70	2.26 ± 9.61	242.74 ± 436.73	5.09 ± 7.11	11.28 ± 22.48
Eicosane	2005.10	1.04 ± 3.16	1354.90 ± 1833.53	12.15 ± 27.11	10.83 ± 11.16
Isopropyl palmitate	2029.80	2.17 ± 9.03	75.63 ± 183.90	8.99 ± 13.25	11.63 ± 32.00
(Z)-9-C18-11-olide	2032.80	4.33 ± 10.11	841.29 ± 1138.35	319.30 ± 450.38	985.96 ± 1393.24
(Z)-9-C18-13-olide	2038.70	221.76 ± 278.84	1933.83 ± 2671.28	544.38 ± 452.76	1779.89 ± 1709.61
12-Octadecanolide	2051.80	-	3.13 ± 7.20	0.54 ± 1.29	0.61 ± 2.44
Macrolide	2056.70	0.8512 ± 2.40	0.19 ± 1.12	-	-
(E)-Octadec-9-en-12-olide	2057.20	-	64.11 ± 79.56	-	25.10 ± 123.85
Macrolide	2058.90	5.31 ± 10.70	11.75 ± 22.80	3.83 ± 5.72	0.55 ± 1.05
Isopropyl_octadecanoate	2063.40	5.57 ± 16.87	277.67 ± 399.88	-	-
(Z9,E11)-C18-13-olide	2069.60	0.06 ± 0.35	1417.08 ± 3035.70	2.66 ± 7.33	165.54 ± 258.28
Octadeca-9-11-dien-13-olide and 11-Octadecanolide	2070.20	-	29.81 ± 101.85	36.17 ± 31.27	47.18 ± 166.82
Isopropyl_linoleate	2073.40	2.53 ± 7.92	929.63 ± 1218.84	-	-
Henicosene	2074.20	28.17 ± 40.01	20.59 ± 25.93	16.14 ± 15.61	327.90 ± 762.56
1-Octadecanol	2081.50	116.92 ± 160.41	67.52 ± 138.34	16.10 ± 32.72	-
Heneicosane	2101.60	2122.79 ± 1879.48	1176.11 ± 2609.26	531.93 ± 392.53	610.33 ± 960.01
Octadecen-18-olide	2123.30	0.27 ± 1.47	15.88 ± 45.96	18.74 ± 29.46	20.29 ± 30.55

17-Octadecanolide	2136.30	-	25.34 ± 143.36	109.12 ± 263.67	149.48 ± 501.09
Isopropyl_octadecadienolate	2130.20	1.67 ± 6.25	99.43 ± 369.98	-	-
9-Octadecen-18-olide	2138.10	44.04 ± 122.04	159.13 ± 217.98	152.97 ± 190.59	190.42 ± 325.61
Octadecanolide	2158.50	-	-	6.56 ± 2.35	-
Ethyl oleate	2165.90	377.55 ± 474.37	606.93 ± 733.31	101.26 ± 172.42	0.34 ± 1.78
Octadecadienolide	2171.70	29.60 ± 71.78	700.86 ± 919.28	598.20 ± 448.49	1413.63 ± 1228.55
Butyl hexadecanoate	2186.90	9.05 ± 13.25	122.77 ± 197.54	28.75 ± 61.82	46.27 ± 79.45
Isopentyl octadecadienoate	2189.30	-	402.28 ± 1692.33	32.41 ± 48.63	25.33 ± 53.00
Isopropyl oleate	2196.10	969.26 ± 828.53	7715.08 ± 6651.53	2070.673 ± 1733.78	3285.38 ± 4207.11
Docosane	2200.90	3.00 ± 7.09	39.28 ± 124.92	6.32 ± 13.64	1.74 ± 4.54
Butyl_octadecanoate	2209.00	17.85 ± 27.85	115.71 ± 162.59	-	-
Eicosanal	2222.30	0.42 ± 2.24	1.05 ± 5.98	0.48 ± 1.60	0.70 ± 3.36
Unknown	2248.70	8.56 ± 20.40	19.21 ± 26.10	0.39 ± 1.29	0.23 ± 1.18
13-Eicosanolide	2252.30	-	6.53 ± 18.44	33.26 ± 62.74	40.95 ± 71.75
Tricosene	2274.90	23.00 ± 33.65	45.26 ± 73.63	19.37 ± 23.37	30.76 ± 45.55
Isobutyl oleate	2297.70	243.89 ± 916.10	883.52 ± 1294.80	146.69 ± 205.08	51.79 ± 163.49
Tricosane	2303.20	168.89 ± 166.76	191.35 ± 273.22	61.88 ± 84.02	59.88 ± 67.54
2-Heneicosanol	2310.60	-	196.39 ± 488.74	7.84 ± 26.02	-
11-Icosenol	2317.40	11.18 ± 19.08	1260.80 ± 4099.99	2.66 ± 6.30	2.09 ± 5.97
Butyl oleate	2359.30	539.28 ± 615.35	2633.57 ± 3949.56	1805.67 ± 1680.41	3411.09 ± 4616.37
Hexenyl hexadecanoate	2379.10	-	12.53 ± 29.20	4.44 ± 10.55	10.27 ± 14.82
Tetracosane	2405.50	15.69 ± 68.45	104.19 ± 513.61	1.60 ± 5.31	-
1_3-Docosanediol	2409.10	0.08 ± 0.32	17.25 ± 42.61	-	-
Macrolide	2417.30	2.324 ± 8.02	2.17 ± 11.45	0.76 ± 1.33	-
Macrolide	2418.70	2.318 ± 3.72	11.22 ± 16.59	19.73 ± 37.04	63.50 ± 97.01
Isoprenyl octadec-11-enoate	2433.60	-	92.19 ± 313.63	-	-
Unknown	2434.60	5.90 ±17.08	17.35 ± 51.73	0.39 ± 1.29	0.23 ± 1.18
(Z)-13-Docosen-1-ol	2461.40	87.02 ± 176.14	297.51 ± 425.23	39.84 ± 63.69	26.27 ± 71.84
Eicosenolide	2475.20	-	-	26.80 ± 44.29	58.17 ± 102.54
1- Docosanol	2488.10	37.55 ± 57.04	140.36 ± 331.25	108.25 ± 126.81	137.70 ± 162.27

Pentacosane	2500.80	136.3 ± 141.58	206.84 ± 803.27	33.49 ± 39.19	30.39 ± 37.85
(Z)-9-Tricosene	2514.30	-	17.94 ± 38.29	-	-
11-methylpentacosane	2532.50	48.70 ± 52.49	7.45 ± 22.8	-	-
Docosen-22-olide	2537.50	-	92.32 ± 117.00	38.40 ± 69.37	-
Hexyl octadecadienoate	2538.70	-	11.25 ± 35.99	7.01 ± 23.26	22.09 ± 37.33
Hexyl octadecenoate and Hexenyl octadecenoate	2553.20	114.56 ± 243.75	679.70 ± 974.09	586.15 ± 580.31	1173.90 ± 1111.75
Hexenyl octadecatrienoate and Hexenyl octadecatrienoate	2555.40	-	16.35 ± 41.97	41.83 ± 96.64	157.85 ± 196.27
Benzyl hexadecanoate	2571.20	-	12.51 ± 26.71	1.20 ± 4.00	1.10 ± 4.26
Hexenyl octadecanoate	2580.30	6.08 ± 16.75	4.99 ± 9.47	1.53 ± 2.67	2.33 ± 5.92
Hexyl octadecanoate	2594.70	0.743 ± 3.93	81.33 ± 95.56	25.69 ± 38.66	68.48 ± 95.79
Hexacosane	2601.10	5.54 ± 11.89	179.42 ± 1012.18	6.31 ± 15.96	-
Tetracosenol	2666.20	205.96 ± 239.41	220.54 ± 319.25	439.32 ± 530.86	405.506 ± 481.61
1-Tetracosanol	2691.80	9.04 ± 21.35	47.67 ± 116.63	138.60 ± 309.57	45.47 ± 73.74
Heptacosane	2700.90	19.83 ± 47.97	225.04 ± 1162.37	98.30 ± 85.46	129.81 ± 143.60
Tetracosenolide	2735.80	2.96 ± 7.36	42.87 ± 56.81	33.63 ± 55.08	59.24 ± 128.91
1,3-Tetracosanediol	2811.90	-	-	58.20 ± 120.24	8.24 ± 19.93
Unknown	2869.20	8.19 ± 19.09	0.29 ± 1.51	3.56 ± 9.01	3.15 ± 16.12
Hexacosanal	2871.80	8.43 ± 28.03	28.60 ± 49.18	-	-
Nonacosane	2901.50	1.57 ± 6.43	167.65 ± 924.25	15.15 ± 19.06	20.82 ± 35.97
13,17-Dimethylnonacosane	2959.20	-	32.82 ± 147.47	43.77 ± 115.55	6.94± 20.82
Cholesterol	3099.40	192.05 ± 273.18	153.25 ± 263.26	617.34 ± 599.42	700.25 ± 647.53
Unknown	3147.40	0.08 ± 0.33	0.68 ± 3.85	6.26 ± 16.19	-
13,17-Dimethylhentriacontane	3158.20	153.09 ± 173.73	395.95 ± 485.06	438.67 ± 328.29	349.94 ± 422.70
2-Nonyl-5-octadecyltetrahydrofuran	3177.50	0.08 ± 0.33	0.20 ± 0.82	22.65 ± 57.78	-
Campesterol or Ergostenol	3207.30	1.42 ± 5.61	7.37 ± 17.36	16.35 ± 21.95	56.38 ± 120.37
13,17-Dimethyltritriacontane	3348.00	24.37 ± 39.54	42.23 ± 105.73	93.10 ± 75.05	64.86 ± 88.28
2-Eicosyl-5-nonyltetrahydrofuran	3370.90	14.77 ± 35.28	20.68 ± 48.38	63.61 ± 50.82	39.84 ± 72.41

Table S8. Probability of mating in no-choice experiments. Hmm: *H. m. malleti*; Htf: *H. t. florencia*; F1: Htf x Hmm; BC: backcrosses [Htf x Hmm] x Htf. Cross type is specified as female x male. [Confidence interval at 95%]. No-choice mating data was collected as in previous studies of *Heliconius* (1,2). This information allowed us to gain a better understanding of the premating barriers operating in this species pair (3). Mating probability for interspecific and hybrid trials was obtained by maximizing the $\log_e$ of the likelihood function (for details see 4,5).

Cross type		N trials	Mating probability	Confidence interval	Source
Control (conspecific)	Htf x Htf	45	0.911	[0.82 - 0.971]	Merot et al. 2017
	Hmm x Hmm	35	0.857	[0.737 - 0.946]	Merot et al., 2017
Interspecific	Hmm x Htf	13	0.152	[0.04 - 0.363]	This study
	Htf x Hmm	16	0.188	[0.157 - 0.377]	This study
Hybrid crosses	**F_1 x Hmm**	**18**	**0**	**[0 - 0.024]**	**Merot et al., 2017**
	**F_1 x Htf**	**24**	**0.249**	**[0.119 - 0.4]**	**Merot et al., 2017**
	Hmm x F_1	8	0	[0 - 0.011]	Merot et al., 2017
	Htf x F_1	10	0.2	[0.04 - 0.45]	Merot et al., 2017
	F_1 x F_1	4	0	[0 - 0.0055]	This study
	**BC x Htf**	**24**	**0.374**	**[0.225 - 0.56]**	**This study**
	**BC x Hmm**	**24**	**0**	**[0 - 0.033]**	**This study**

Figure S1. Map showing the geographic distribution and wing phenotype of *H. melpomene malleti* and *H. timareta florencia*.

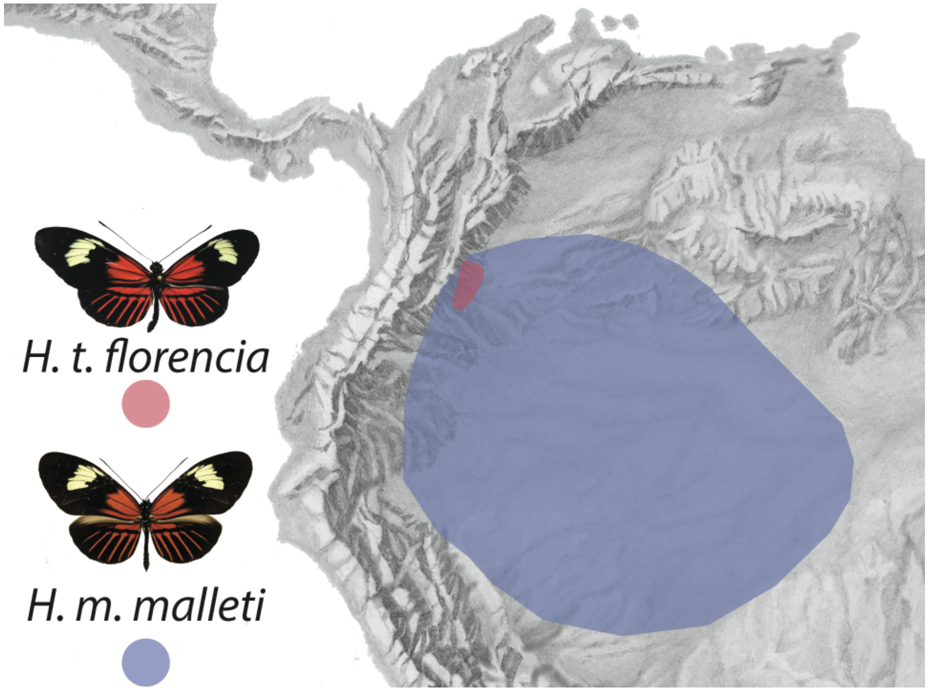

Figure S2. Location of landmarks (LM) coordinates on the forewings and hindwings of *H. melpomene malleti* and *H. timareta florencia*. (A) LM used in the colour pattern analysis, (B) LM used in the shape and size analyses. Shape deformation (C) and (D) represents the shape at minimum values for PC1 and PC2, respectively. Shape deformation (E) and (F) represent maximum values for PC1 and PC2, respectively. LM3, LM4 and LM15 are those that vary the most.

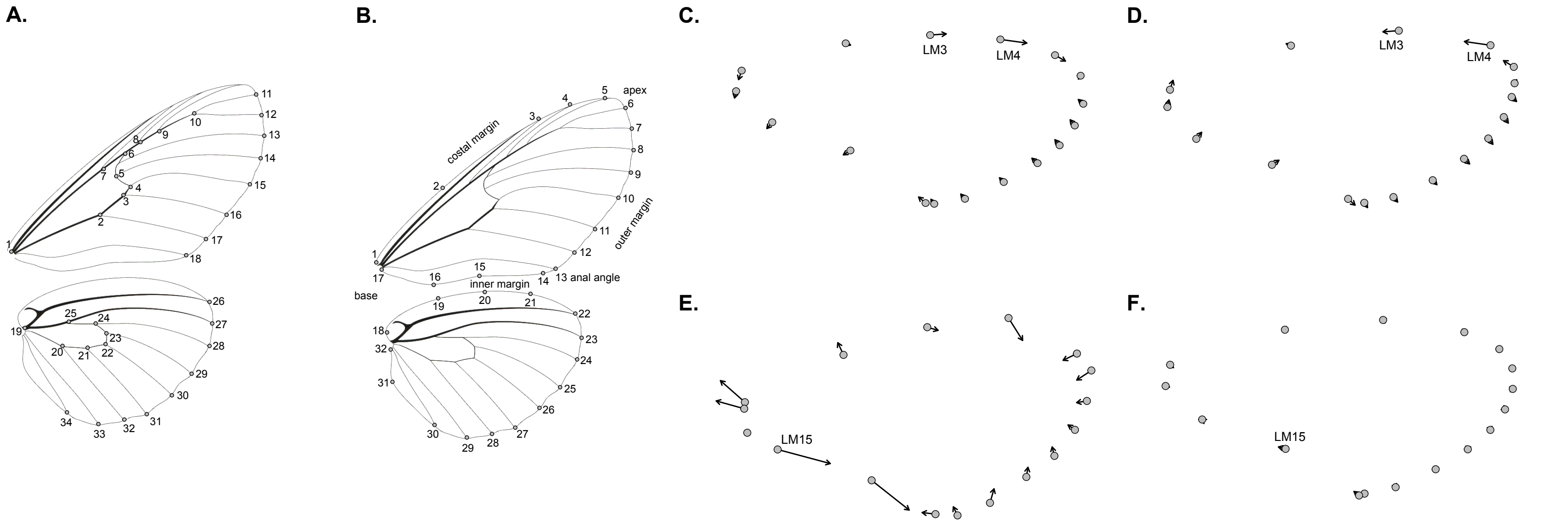

Figure S3. Species wing size and shape. Forewing (A) and hindwing (B) size variation. Density plots showing the variation in the shape of the forewing (C) and the hindwing (D). A total of 43 *H. melpomene malleti* and 45 *H. timareta florencia* were analysed.

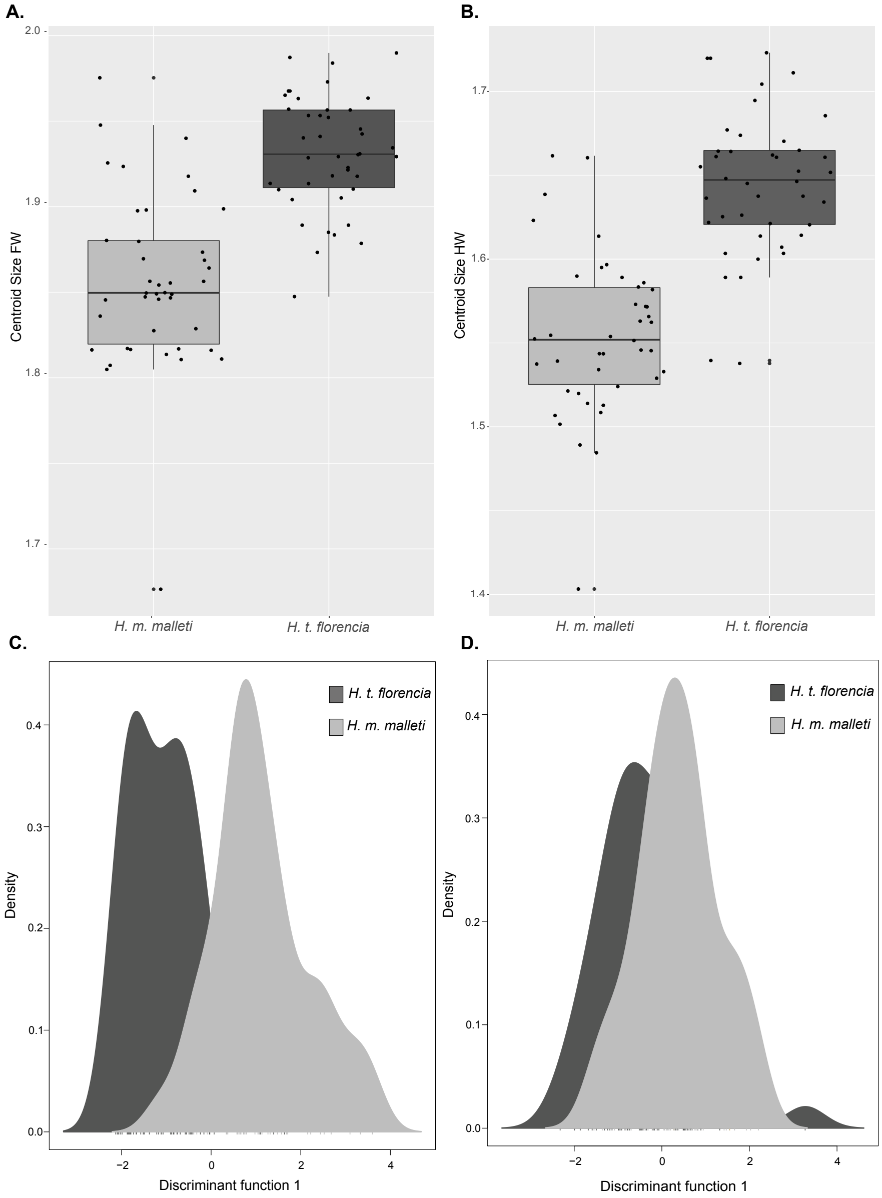

Figure S4. (A) Shape variation of forewings of *H. melpomene malleti* (light grey) and *H. timareta florencia* (dark grey). (B) and (C) show the PC1 and PC2 loadings, respectively.

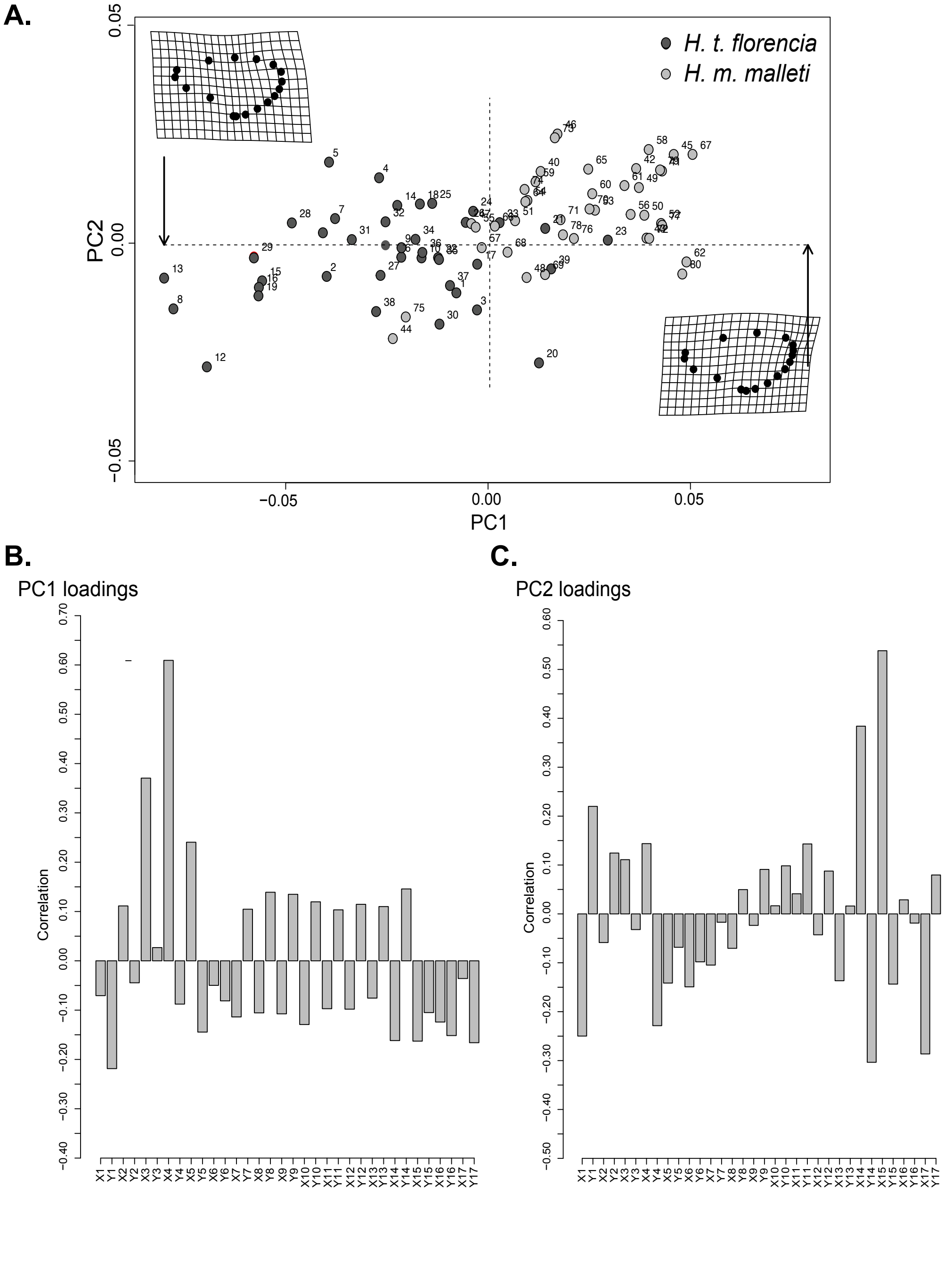

Figure S5. Colour pattern comparison between the two species. (A) Yellow patch on the dorsal forewing ($F_{(1,80)}$=0.0647, p>0.01); (B) Dennis on the dorsal forewing ($F_{(1,79)}$=0.0603, p>0.01); (C) Ray on the dorsal hindwing ($F_{(1,79)}$=0.5929, p>0.01); (D) Yellow patch on the ventral forewing ($F_{(1,74)}$=0.6191, p>0.01); (E). Dennis on the ventral forewing ($F_{(1,74)}$=1.2597, p>0.01) and (F) ray on the ventral hindwing ($F_{(1,74)}$=0.6584, p>0.01). Only individuals with wings in good condition were used (n=88; Table S1). Yellow circles = *H. t. florencia.* Red triangles = *H. m. malleti*. The Δ colour scale indicates how present the colour pattern is, where positive values (red) represent higher presence and negative values (blue) represent lower presence or even absence. PC1 explains the variation in size and PC2 explains the variation in the shape of said colour pattern element.

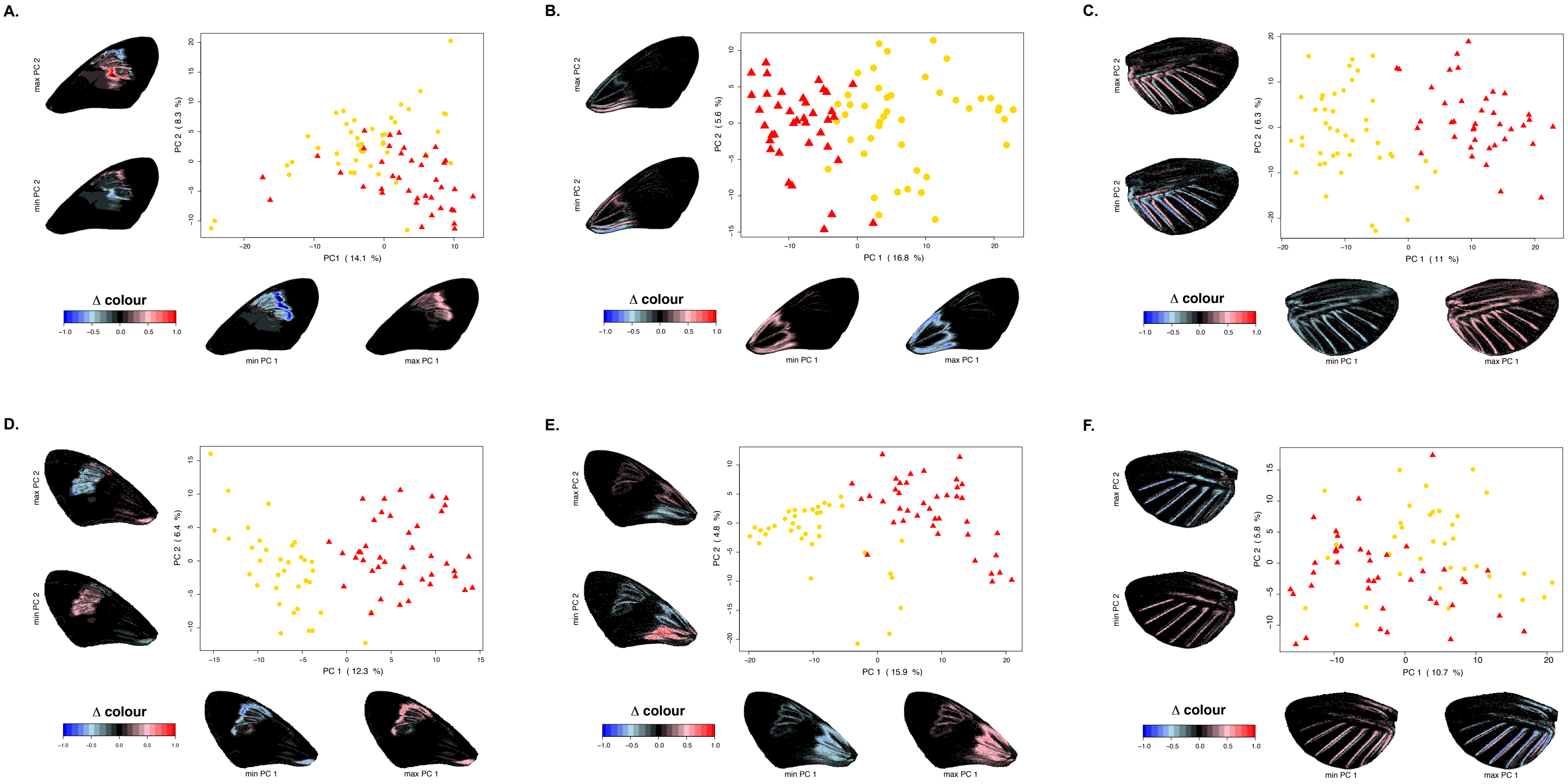
A.
B.
C.
D.
E.
F.
max PC 2
min PC 2
PC1 (14.1 %)
PC 2 (8.3 %)
PC 1 (16.8 %)
PC 2 (5.6 %)
PC 1 (11 %)
PC 2 (6.3 %)
PC 1 (12.3 %)
PC 2 (6.4 %)
PC 1 (15.9 %)
PC 2 (4.8 %)
PC 1 (10.7 %)
PC 2 (5.8 %)
Δ colour
−1.0 −0.5 0.0 0.5 1.0
min PC 1
max PC 1

Figure S6. Mate choice triads testing the importance of wing colour pattern in mate preference. The number of matings obtained is indicated above each bar. Control males are represented in light grey and treatment males are represented in dark grey. (exact binomial test p=0.55 in both cases).

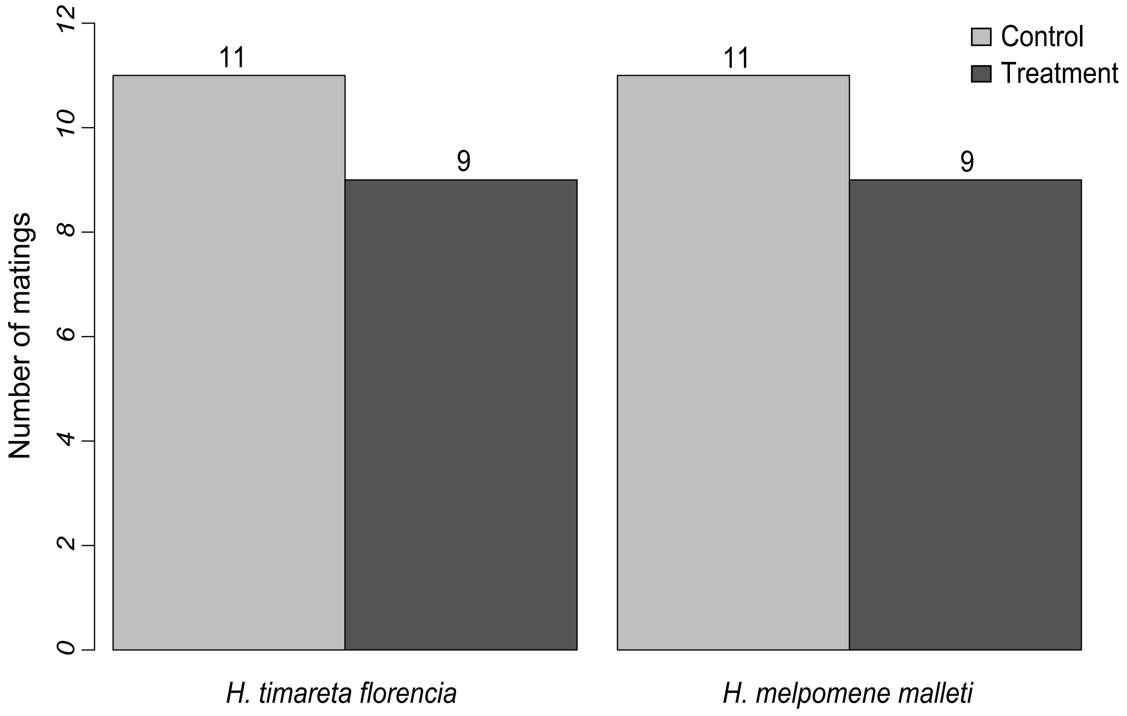

Figure S7. Amount (ng) of compounds that remained in the wings of perfumed males before evaporation. We quantified the presence of the perfume applied at the beginning of the experiment, at 1 minute, at 30 minutes and at 60 minutes after spreading perfume. (A) Octadecanal; (B) Heneicosane; (C) Syringaldehide; (D) Z-11-eicosanal.

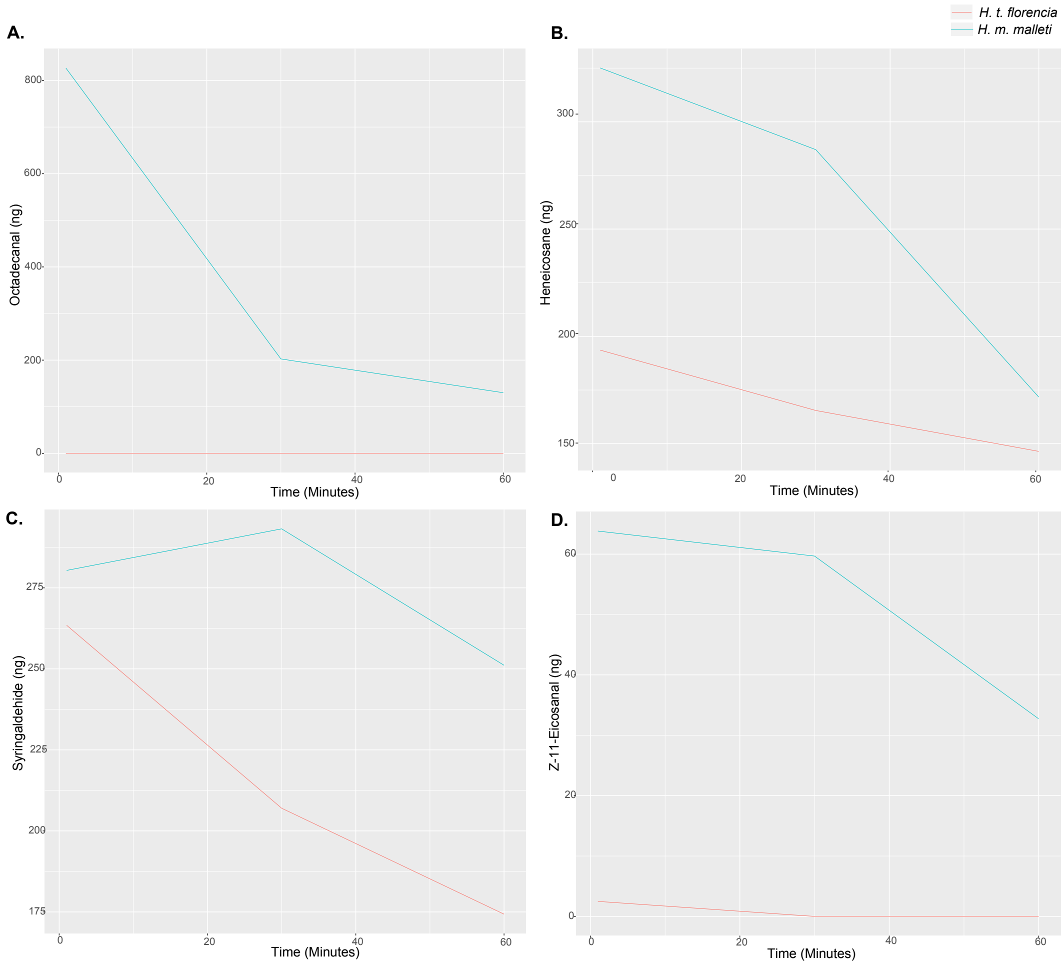

Figure S8. Mate choice triads testing behavioural responses in F1 and backcross (BC) females. (A). The number of matings obtained is indicated above each bar. (B) Proportion of courtships that resulted in behavioural responses in F_1 and backcross (BC) females. Behaviours recorded were acceptance (A) or rejection (R) towards males of *H. m. malleti* (red, left) and *H. t. florencia* (blue, right). Means are marked with a black square and boxplots mark the inter-quartile ranges. Size of datapoint is proportional to the number of courtships by that male. The asterisk (*) next to the female (F_1/BC) is indicative of statistical significance (α=0.01) according to GLMM.

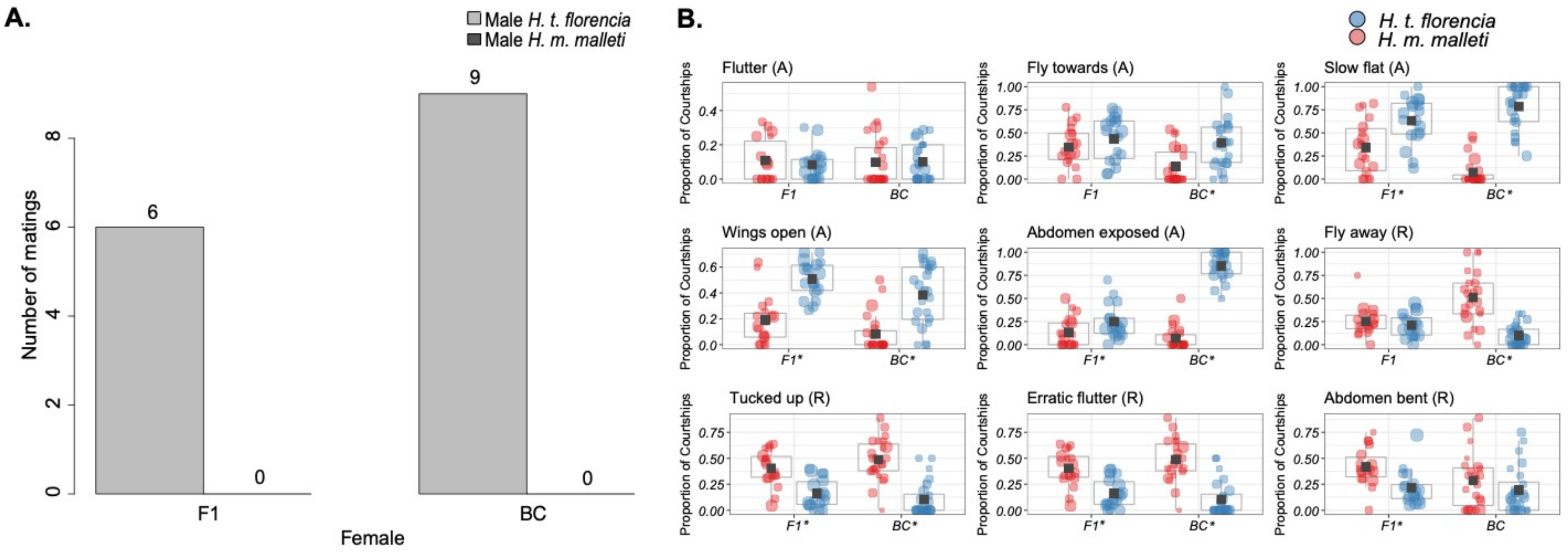

Figure S9. Species differences in male androconia extracts. Chromatogram of extract of androconial region from (A) *H. timareta florencia* and (B) *H. melpomene malleti*. IS, internal standard (2-tetradecylacetate); 1, dihydroactinidiolide; 2, unknown; 3, syringaldehide; 4, henicosane; 5, octadecanal.

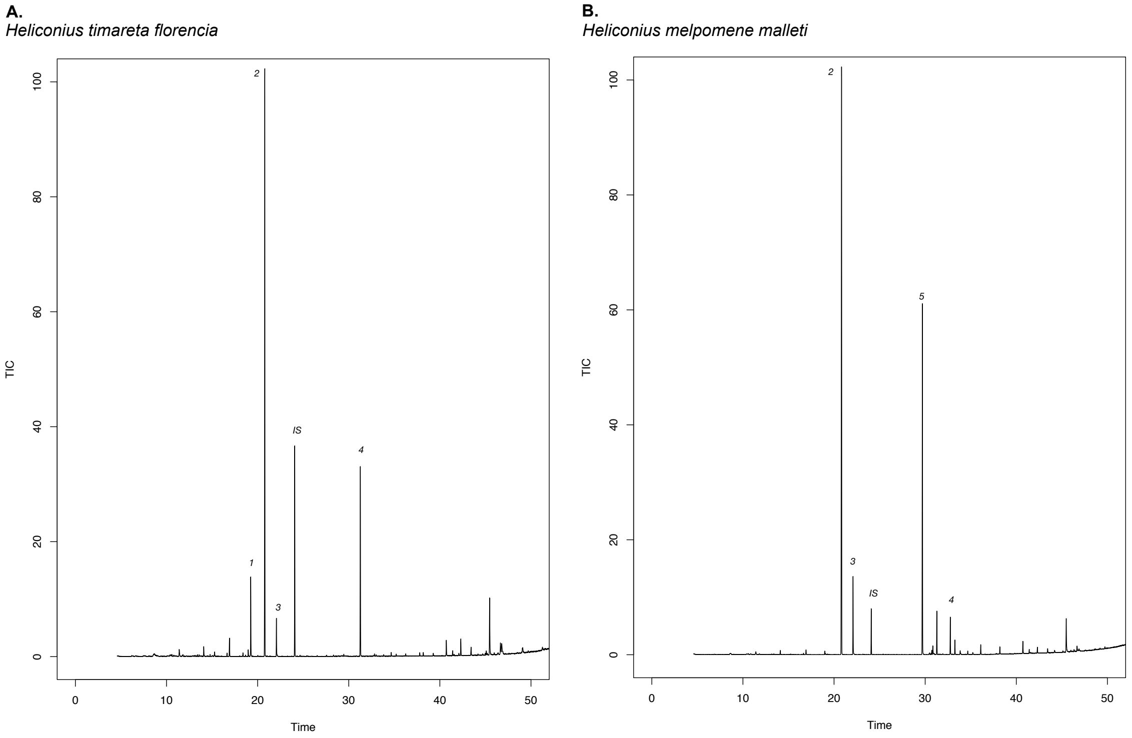

Figure S10. Cluster analysis based on Euclidian distance of compound composition in the wing androconia of males of *H. melpomene malleti*, *H. timareta florencia*, F_1 and backcrosses (BC). Compounds highlighted in red are the most abundant.

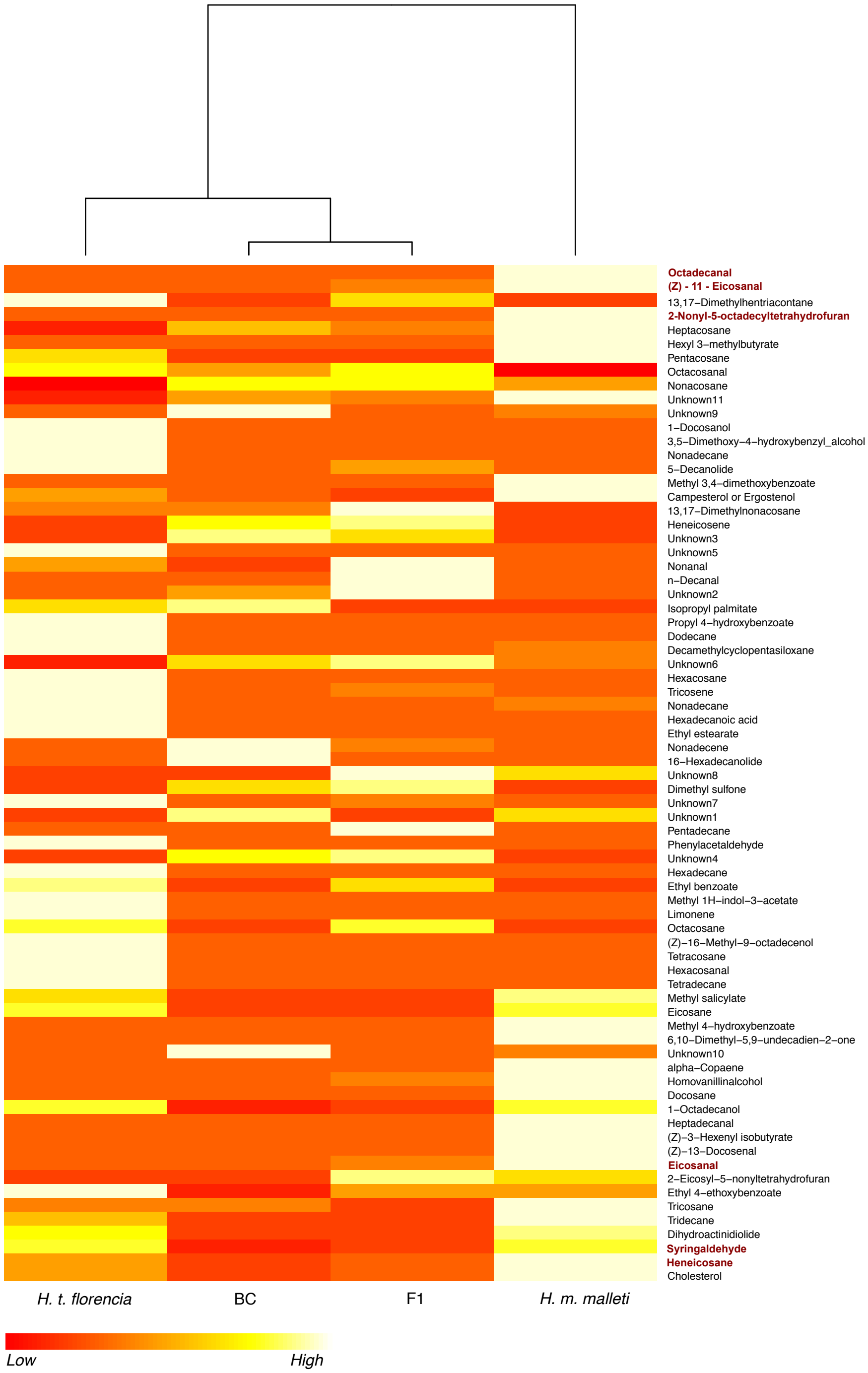

Figure S11. Chromatogram patterns obtained from androconial extracts of F_1 and backcross males. (A) F_1 individuals and (B) backcross individuals. IS, internal standard (2-tetradecylacetate); 1, dihydroactinidiolide; 2, unknown; 3, syringaldehide; 4, henicosane; 5, octadecanal.

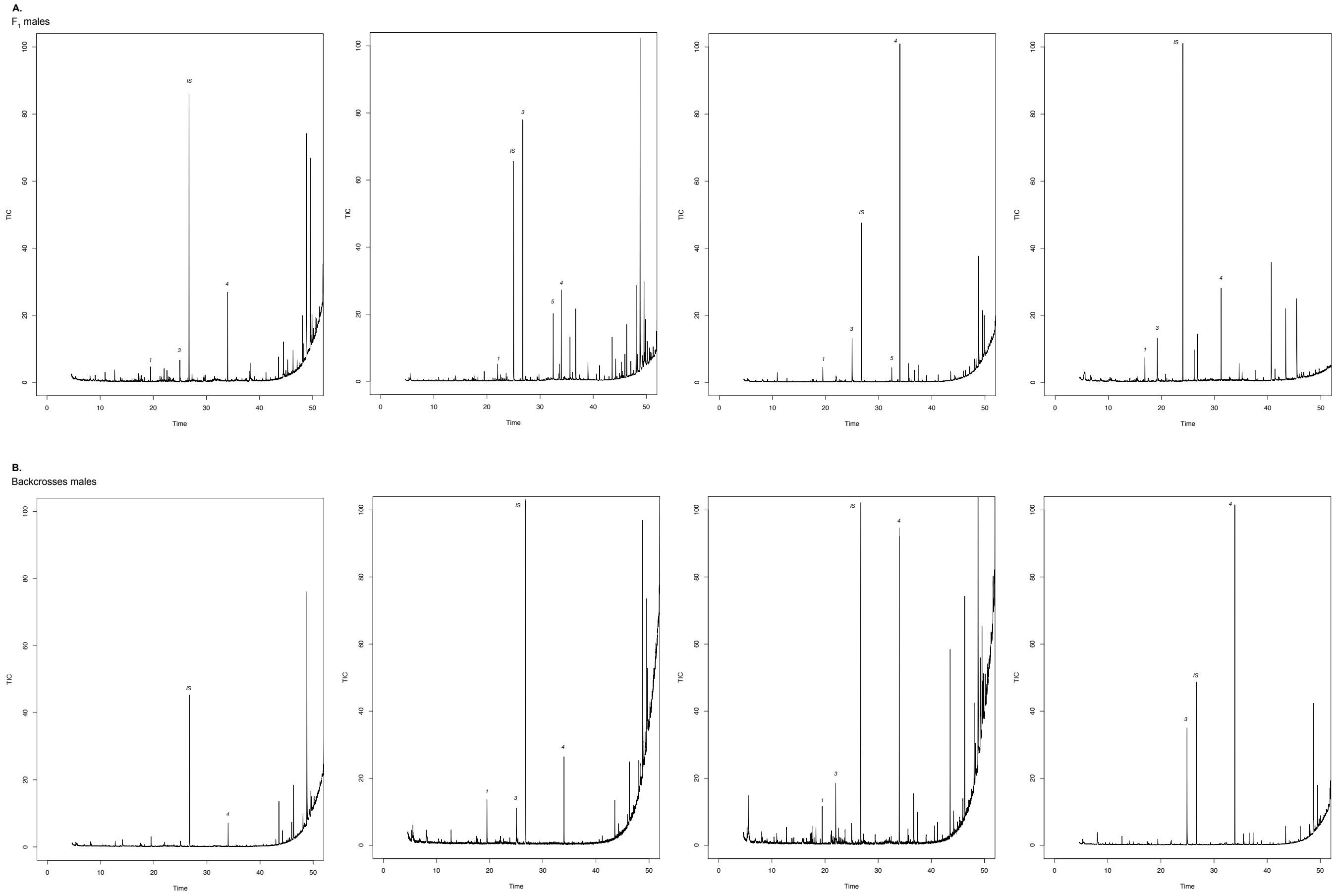

Figure S12. Chromatogram pattern of the abdominal gland bouquet of males. (A) *H. timareta florencia*. (B) *H. melpomene malleti*. IS, internal standard (2-tetradecylacetate); 1, eicosane; 2, Z-9-C18,11olide; 3, heneicosane; 4, ethyl oleate; 5, isopropyl oleate; 6, isopropyl octadecanoate; 7, butyl oleate; 8, β-ocimene; 9, henicosene.

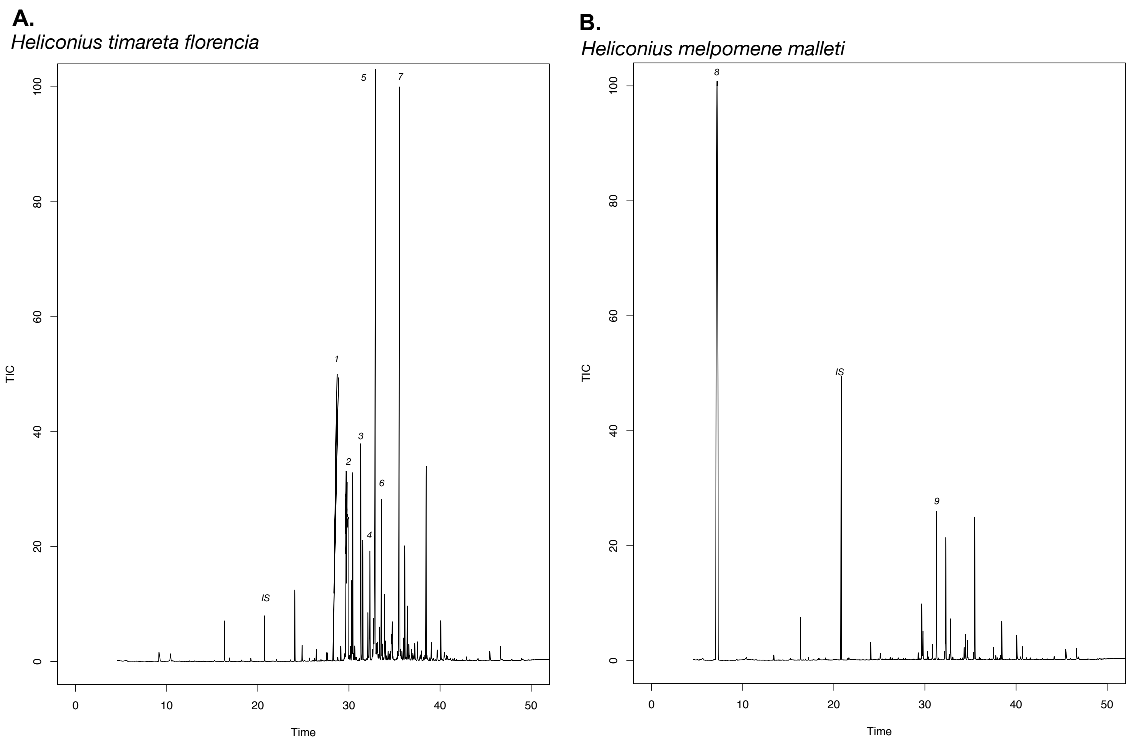

Figure S13. Cluster analysis based on Euclidian distance of the compound composition of the wing androconia of males of *H. melpomene malleti*, *H. timareta florencia*, F_1 and backcrosses (BC). Compounds highlighted in red are the most abundant.

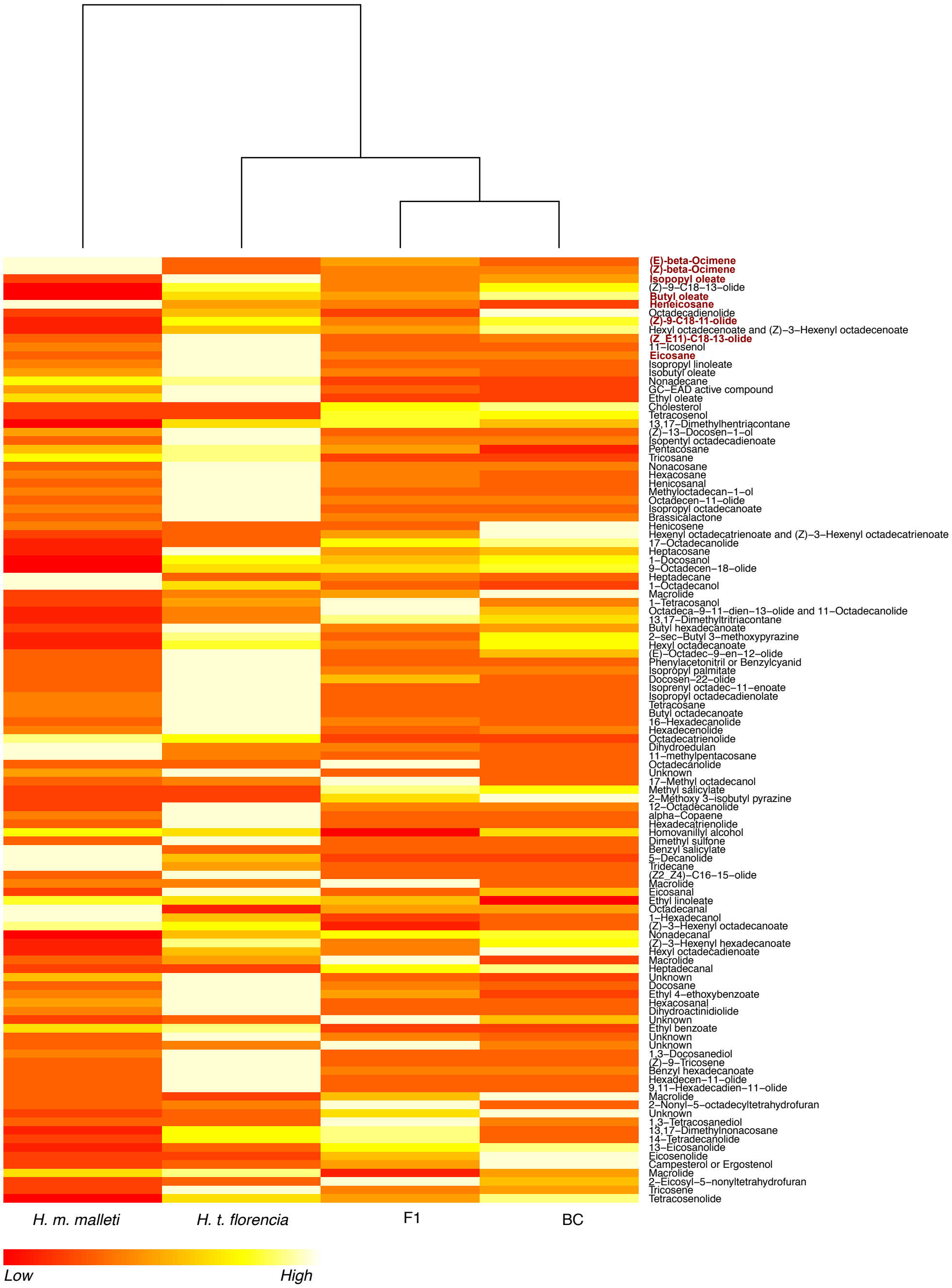

Figure S14. Chromatogram patterns obtained from abdominal gland bouquet of F_1 and backcross males. (A) F_1 individuals and (B) backcrosses individuals. IS, internal standard (2-tetradecylacetate); 1, eicosane; 2, Z-9-C18,11olide; 3, heneicosane; 4, ethyl oleate; 5, isopropyl oleate; 6, isopropyl octadecanoate; 7, butyl oleate; 8, β-ocimene; 9, henicosene.

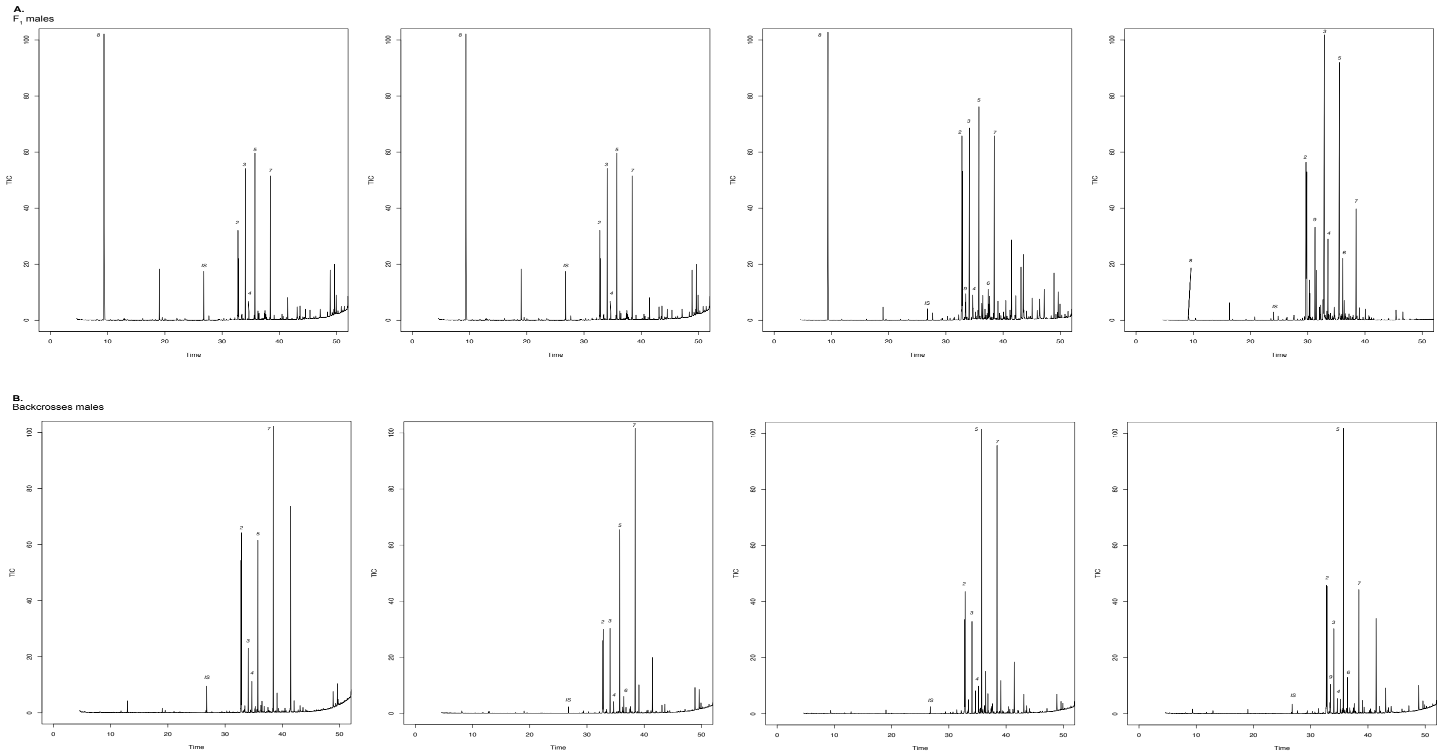

REFERENCES

1. Naisbit RE, Jiggins CD, Linares M, Salazar C, Mallet J. Hybrid Sterility, Haldane's Rule and Speciation in Heliconius cydno and H. melpomene. Genet Soc Am. 2002;161:1517–26.
2. Muñoz AG, Salazar C, Castaño J, Jiggins CD, Linares M. Multiple sources of reproductive isolation in a bimodal butterfly hybrid zone. J Evol Biol. 2010;23(6):1312–20.
3. Mérot C, Salazar C, Merrill RM, Jiggins C, Joron M. What shapes the continuum of reproductive isolation? Lessons from Heliconius butterflies. Proc R Soc B Biol Sci. 2017;284:20170335.
4. Mérot C, Frèrot B, Leppik E, Joron M. Beyond magic traits: Multimodal mating cues in Heliconius butterflies. Evolution (N Y). 2015;69(11):2891–904.
5. Sanchez AP, Pardo-Diaz C, Enciso-Romero J, Munoz A, Jiggins CD, Salazar C, et al. An introgressed wing pattern acts as a mating cues. Evolution (N Y). 2015;69(6):1619–29.
